# One-pot synthesis of bimetallic Fe–Cu metal–organic frameworks composite for the elimination of organic pollutants via peroxymonosulphate activation

**DOI:** 10.1007/s11356-023-30026-5

**Published:** 2023-10-18

**Authors:** Antía Fdez-Sanromán, Emilio Rosales, Marta Pazos, Angeles Sanromán

**Affiliations:** Department of Chemical Engineering, CINTECX, Universidade de Vigo, Campus Universitario As Lagoas—Marcosende, 36310 Vigo, Spain

**Keywords:** Advanced oxidation processes, Fenton-like, Metal–organic framework, Response surface methodology, Sulphate and hydroxyl radicals, Rhodamine B removal

## Abstract

A series of bimetallic of FeCu metal–organic frameworks (MOFs) have been synthesised using a solvothermal process by varying the ratio between the two metals. Further, the bimetallic MOF catalysts were characterised by X-ray powder diffraction, scanning electron microscopy, and infrared spectroscopy techniques. Their catalytic properties for activation of peroxymonosulphate (PMS) have been tested by the removal of a model dye, rhodamine B. As a result, NH_2_–Fe_2.4_Cu_1_-MOF demonstrated the highest degradation, the effect of the ratio NH_2_–Fe_2.4_Cu_1_-MOF/PMS has been studied, and the main reactive species have been assessed. The application of these MOFs in powder form is difficult to handle in successive batch or flow systems. Thus, this study assessed the feasibility of growing NH_2_–Fe_2,4_Cu_1_-MOF on polyacrylonitrile (PAN) spheres using the one-pot solvothermal synthesis method. The optimisation of the catalytic activity of the synthesised composite (NH_2_–Fe_2.4_Cu_1_-MOF@PAN) has been evaluated by response surface methodology using a central composite face-centred experimental design matrix and selecting as independent variables: time, PMS concentration, and catalyst dosage. Based on the results, the optimisation of the operational conditions has been validated. At 2.5 mM PMS, 90 min, and 1.19 g·L^−1^ of catalyst dosage, maximum degradation (80.92%) has been achieved, which doubles the removal values obtained in previous studies with other MOFs. In addition, under these conditions, the catalyst has been proven to maintain its activity and stability for several cycles without activity loss.

**Graphical Abstract:**

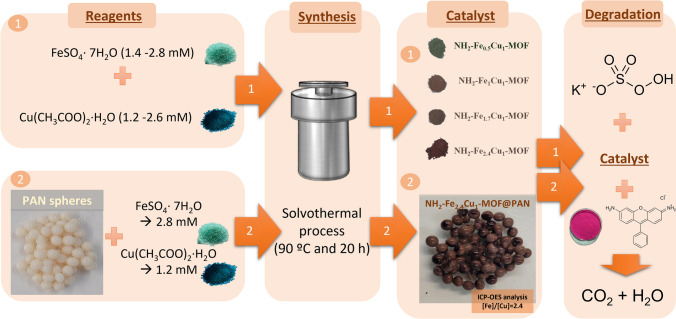

## Introduction

In the last decade, the effects of climate change and environmental pollution have become a reality, affecting the availability of water and new alternative sources are required. The UN global sustainable development goal 6 of clean water and sanitation includes as a target the improvement of water quality, wastewater treatment, and safe reuse (United Nations General Assembly 2015). One of these solutions is the treatment of polluted waters for reuse in different industrial or agricultural sectors. A significant portion of these polluted waters are originated from industry, particularly the textile and pharmaceutical industries. In these sectors, the production of dyes is very high and this means a high direct discharge of these organic compounds into the water (Wang et al. 2023b). In addition, water treatment plants are normally not able to completely remove these pollutants and generate large volumes of sludge, resulting in secondary pollution (Mahmoodi et al. 2019). For this reason, in the last decade, scientists have developed treatment techniques to remove these persistent pollutants. Among these techniques, the advanced oxidation processes (AOPs) stand out.

AOPs are based on the in situ production of non-selective radicals with a high oxidation power and able to degrade recalcitrant compounds. This fact allows radicals to easily break the bonds that form the organic molecule of the pollutant until it has completely mineralised. Of these radicals, sulphate and hydroxyl radicals are the essential active radicals in AOPs. Among them, it should be noted that sulphate radicals have a higher oxidative capacity, 2.5–3.1 V versus 1.8–2.7 V from hydroxyl radical (Liu et al. 2022a); longer lifetimes, 30–40 μs against 1 μs, and work in a wider pH range (Zhang et al. 2022a). For their generation, the uses of peroxymonosulphate (PMS) and persulphate (PS) in Fenton-like processes have attracted significant attention and are feasible alternatives for AOP processes (Wang and Wang 2018). PMS can be activated to generate sulphate and hydroxyl radicals by heat (Xu et al. 2019b), ultrasound (Pang et al. 2019), electric field (Zuo et al. 2022), light (Pham et al. 2021), and transition metals (Kohantorabi et al. 2021). Activation by transition metals is the most commonly used option and Co, Cu, Fe, or Ni are normally selected as catalysts (Cherifi et al. 2019). Co is the one that best activates the formation of sulphate radicals, but several concerns have arisen about its toxicity. Fe is a good activator and the most eco-friendly and economical of the transition metals (Liu et al. 2022b). Cu is normally also used to replace Co because of its good activating power and because it is less toxic (Liu et al. 2022a). All the metals mentioned before can be found as homogeneous or heterogeneous catalysts. The reactions that take place to produce sulphate radicals with metals (Me^n^) as catalysts of PMS (Eq. 1) and PS (Eq. 2) are as follows (Wang and Wang 2018):

1 $${\mathrm{HSO}}_{5}^{-}+{Me}^{n}\to {Me}^{n+1}+{SO}_{4}^{\bullet -}+{\mathrm{OH}}^{-}$$

2 $${S}_{2}{O}_{8}^{-2}+{Me}^{n}\to {Me}^{n+1}+{SO}_{4}^{\bullet -}+{SO}_{4}^{-2}$$

However, there are several limitations for the homogenous systems (Wang and Wang 2018): i) difficult to recovery the metal ions; ii) for the wastewater containing high amounts of organic pollutants, high amounts of metal ions are required, which results in the existence of large amounts of metal ions in the effluent; iii) the species of transition metals are highly influenced by the pH and composition of the water. For this reason, the homogeneous catalyst is now disused since heterogeneous catalysts provide meaningful advantages. These advantages include the easy recovery of the catalyst and the ability to operate in both batch and flow systems (Zhang et al. 2022b). Among the heterogeneous catalysts, metal–organic frameworks (MOFs), which are a class of porous, crystalline materials with a broad range of applications that have been reported in the literature (Xu et al. 2019a; Fdez-Sanromán et al. 2022b). Due to their excellent properties, they are widely utilised in a variety of applications, such as biomedicine (Sharabati et al. 2022), environmental treatment (Petit 2018; Fdez-Sanromán et al. 2022a), or energy and gas storage (Boorboor Ajdari et al. 2020). These MOFs were initially synthesised with a single metal at the centre of the structure. Nowadays, some studies reinforce the catalytic properties of materials by adding one or more metals to the structure. Bimetallic MOFs include two metals coordinated with an organic linker and their chemical properties are due to synergistic effects (Chen et al. 2020). Furthermore, secondary metal incorporation onto MOF surfaces has been associated with high porosity, better thermal stability, enhanced intrinsic properties, enhanced electronic conductivity, and increased active site availability compared to monometallic MOF surfaces (Zhou et al. 2022). Even though it has a more complex structure, the most common method of synthesis is the same as the monometallic MOF. Thus, it requires three components: metal salts (in this case two different metals), the organic linkers, and the solvent, in combination with different temperatures and pressures (Pandey 2021). In this case, the reaction between ligands and two metal ions of nearly the same electronic configuration and charge density results in a bimetallic MOFs that form a single phase instead of a combination of two monometallic compounds (Raza et al. 2021). In light of these characteristics, bimetallic MOFs appear to be promising catalysts for AOP processes, and it is of interest to explore ways to improve their ability to be retained and reused. To overcome the limitation of small catalysts, in this study, a new method in which the bimetallic MOF is grown on the polyacrylonitrile (PAN) spheres has been designed to enhance the scalability, mechanically robustness, and reusability of the catalyst.

The aim of this work is to synthesise a bimetallic MOF with high catalytic capacity and its immobilisation in spheres for its application in batch/continuous treatment systems. To the best of our knowledge, this paper is the first to study the synthesis of FeCu-MOF on the support material such as PAN sphere. Thus, first, different Fe and Cu ratios have been used in the synthesis of bimetallic MOFs by means of a solvothermal process, and their catalytic capacity is studied by the elimination of a model contaminant, rhodamine B, using PMS as oxidant. The best catalyst in terms of activity capacity is then grown on PAN spheres, evaluated as a supported catalyst and the optimisation of the process is carried out. The influence of the reaction time, the concentration of the catalyst, and PMS are crucial factors. Therefore, in this study, the optimisation of these factors is made by response surface methodology (RSM) using a central composite face-centred design (CCF-CD). Lastly, under the optimum conditions obtained, its viability in continuous processes is evaluated, by means of its reuse in successive cycles.

## Materials and methods

### Chemicals and material

For the synthesis of the NH_2_–Fe_x_Cu_y_-MOF and NH_2_–Fe_2.4_Cu_1_-MOF@PAN, the used reagents were PAN (100%), dimethylformamide (DMF, ≥ 99.8%), 2-aminoterephthalic acid (NH_2_BDC, 97%), ethanol (EtOH, ≥ 99.5%), copper (II) acetate (Cu(CH_3_COO)_2_⋅H_2_O, 98%), and iron (II) sulphate heptahydrate (FeSO_4_·7H_2_O, 99%). All the chemicals mentioned were purchased from Sigma-Aldrich. The model dye used, rhodamine B (100%), and the PMS (2KHSO_5_·KHSO_4_·K_2_SO_4_, 100%) were supplied also by Sigma Aldrich. For the determination of specific reactive oxygen species (ROS), EtOH (≥ 99.5%), tert-butanol (TBA, ≥ 99%), and p-benzoquinone (p-BQ, ≥ 98%) were used. These three reagents were supplied by Sigma Aldrich.

All experimental solutions were prepared using ultrapure deionised water.

### Synthesis of NH_2_–Fe_x_Cu_y_-MOF

**Fig. 1:**
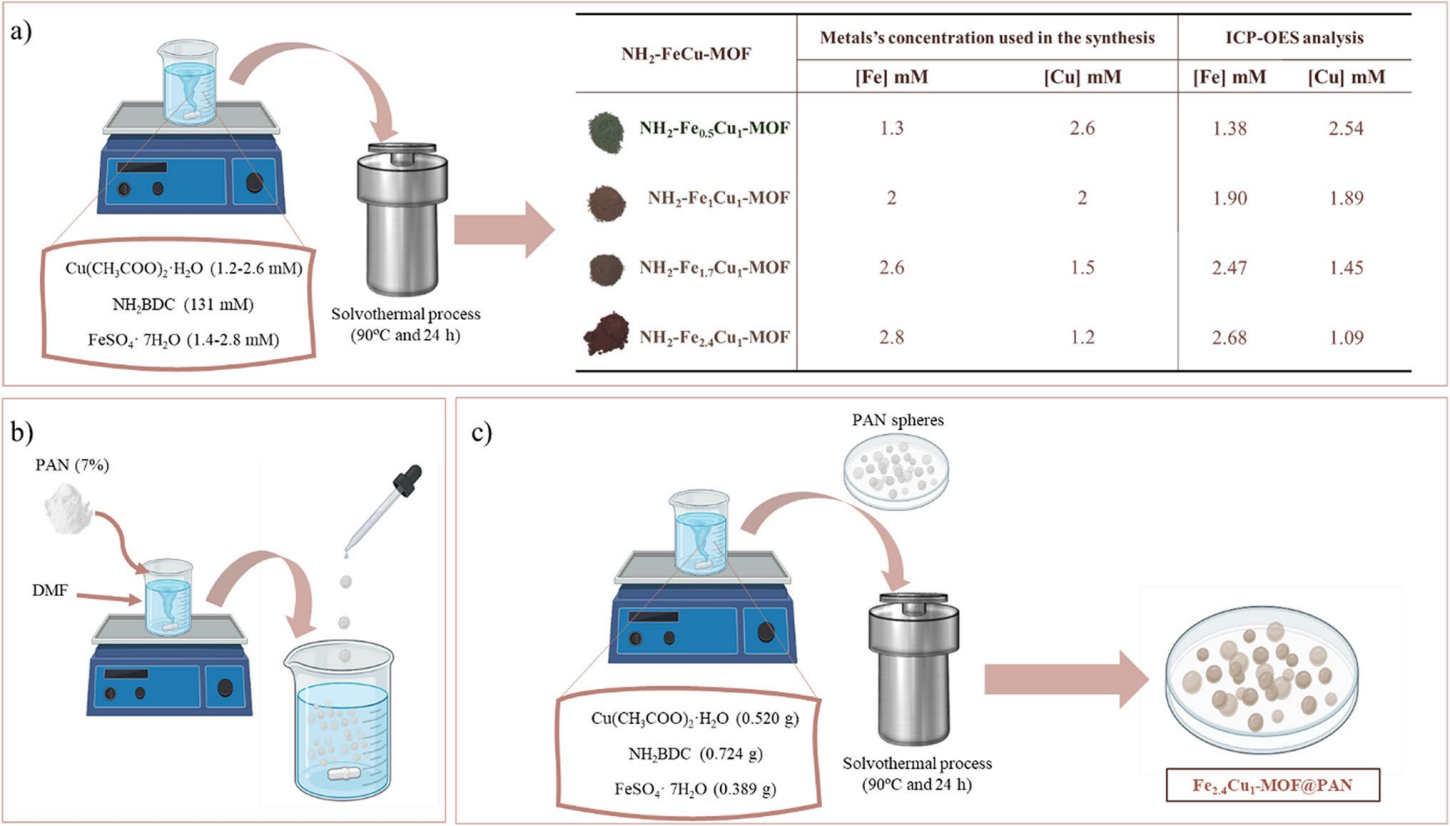
Graphical representation of the synthesis processes of **a** the four NH_2_–Fe_x_Cu_y_-MOFs with the metals concentration used and the ICP-OES analysis after synthesis, **b** PAN spheres, and **c** NH_2_–Fe_2.4_Cu_1_-MOF@MOF-PAN

The synthesis procedure of various NH_2_–Fe_x_Cu_y_-MOF was based on the reported by Fu et al. (2022) with slight modifications. For the synthesis of NH_2_–Fe_0.5_Cu_1_-MOF, 0.724 g of NH_2_BDC has been mixed for 15 min in 32 mL of DMF. Subsequently, 0.389 g of FeSO_4_·7H_2_O and then 0.520 g Cu(CH_3_COO)_2_⋅H_2_O have been added. While adding these metal salts, 2 mL of ethanol and 2 mL of water have been poured in. After homogenising the mixture for 60 min, the solution is transferred to a 100-mL autoclave Teflon reactor, in which it is kept at 90 °C for 20 h in an oven (Fig. 1a). The obtained material has been cleaned two times with DMF and three times with ethanol and then has been dried in an oven at 80 °C. A similar procedure has been followed for the synthesis of the other MOFs (NH_2_–Fe_1_Cu_1_-MOF, NH_2_–Fe_1.7_Cu_1_-MOF and NH_2_–Fe_2.4_Cu_1_-MOF), which are differentiated by the ratio Fe:Cu metals. For the synthesis of all NH_2_–Fe_x_Cu_y_-MOFs, it has always been maintained that the total sum of both metals was 4 mmol.

### Synthesis of NH_2_–Fe_2.4_Cu_1_-MOF@PAN

As a first step in the synthesis of NH_2_–Fe_2.4_Cu_1_-MOF@MOF-PAN, spheres of PAN have been synthesised by a droplet method (Fig. 1b). For this, PAN (8%) has been first dissolved in DMF with vigorous stirring in a 50 °C water bath for 2 h. Then, to facilitate the formation of the PAN sphere, as reported by Riley et al. (2020), the mixture has been placed in a syringe and dripped into cold distilled water, at a temperature between 2 and 5 °C, with gentle agitation. Finally, several washes with distilled water have been made to ensure that no DMF remains. Once these PAN spheres were obtained, the synthesis of NH_2_–Fe_2.4_Cu_1_-MOF was carried out as indicated in the previous section, except that these spheres must be added to the solution before adding it to the hydrothermal reactor (Fig. 1c).

### Fenton-like PMS process

In all the experiments with NH_2_–Fe_x_Cu_y_-MOF and NH_2_–Fe_2.4_Cu_1_-MOF@PAN, the degradation of the rhodamine B at the initial concentration of 10 mg·L^−1^ has been performed on a volume of 50 mL. In the assays to evaluate the catalytic capacity and select the best alternative of NH_2_–Fe_x_Cu_y_-MOF, a concentration of 1 mM of PMS and 0.25 g·L^−1^ of catalyst have been employed. With the most effective catalyst, different concentrations of PMS (1, 2, and 2.44 mM) and catalyst (0.25 and 0.5 g·L^−1^) were evaluated.

In all experiments, samples were taken periodically to determine the rhodamine B removal (“Quenching assays” section).

### Quenching assays

To better understand the degradation mechanism of rhodamine B, the effects of ROS in the dye degradation have been determined. To do that, performing masking experiments, in the same working conditions as descripted in the “Fenton-like PMS process” section using the best NH_2_–Fe_x_Cu_y_-MOF have been carried out. EtOH, TBA, and p-BQ have been used due to they react with sulphate (SO_4_^•−^), hydroxyl (OH^•^), and superoxide (O_2_^•−^) radical species, respectively. To ensure that all the species to be analysed have reacted, the concentrations of EtOH (300 mM), TBA (500 mM), and p-BQ (20 mg·L^−1^) are high.

### Analytical procedures

Sample aliquots of 1 mL have been withdrawn at predetermined time intervals and the residual rhodamine B concentration has been measured through a UV–Vis spectrophotometer (Thermo Fisher Genesys M-150) at 554 nm. All the results are the average of duplicated assays, and the standard deviations were below 5%.

### Characterisation of NH_2_–Fe_x_Cu_y_-MOF and NH_2_–Fe_2.4_Cu_1_-MOF@PAN

To determine the correct synthesis of the different NH_2_–Fe_x_Cu_y_-MOF and NH_2_–Fe_2.4_Cu_1_-MOF @PAN, the material characterisation has been performed by several techniques. Fourier-transform infrared spectroscopy (FTIR) analysis has been carried out with the Nicolet 6700, Thermo Fisher Scientific Inc. equipment. X-ray diffraction (XRD) analysis has been made on a Siemens D5000 diffractometer, and the determination of the metal content was carried out by inductively coupled plasma-optical emission spectrometry (ICP-OES), using a Thermo Scientific™ iCAP™ PRO XP DUO. In addition, scanning electron microscopy and energy dispersive spectrometry (SEM/EDS) using a JEOL JSM6010LA with EDS Oxford AZtecOne SEM has been used to analyse the surface of the different materials. ImageJ analysis software was used to determine the average diameter of each NH_2_–Fe_x_Cu_y_-MOF based on 20 measurements. All the equipment mentioned in this section belongs to the Centro de Apoio Científico-Tecnolóxico á Investigación (C.A.C.T.I.) from the University of Vigo (Vigo, Spain).

For the measurement of the specific surface area, Brunauer–Emmett–Teller (BET) has been evaluated by N_2_ adsorption–desorption isotherm using a MicroActive TrisStar ® II PLUS instrument, that belongs to Servizos de Apoio á Investigación (SAI) from Universidade da Coruña (A Coruña, Spain).

### Experimental design to optimise the Fenton-like PMS process using NH_2_–Fe_2.4_Cu_1_-MOF@PAN

A response surface methodology with a central composite face-centered (CCF) experimental design matrix has been applied to design the experiments. Under this methodology, it is possible to identify optimal conditions while minimising the number of experiments required for a selected response. To optimise the Fenton-like PMS process using NH_2_–Fe_2.4_Cu_1_-MOF@PAN, three operating parameters have been investigated: time (*X*_1_), NH_2_-Fe_2.4_Cu_1_-MOF@PAN dosage (*X*_2_), and PMS (*X*_3_) as factors that may potentially affect the response function, which is dye decolourisation. Also, these independent variables were studied at two levels (+ 1 and − 1). The assignments of these levels for each of the variables and the related actual values are shown in Table 1.

**Table 1:** Actual and coded levels of the independent variables (*X*_1_, *X*_2_, and *X*_3_) for the selected response, rhodamine B elimination

Coded level	*X*_1_, time (min)	*X*_2_, NH_2_–Fe_2.4_Cu_1_-MOF@PAN dosage (g·L^−1^)	*X*_3_, PMS concentration (mM)
− 1	10	0.25	1.5
0	50	0.75	2
+ 1	90	1.25	2.5

The matrix design resulted in a total of 20 experiments, including six replicates at the central point, to optimise the Fenton-like process of rhodamine B degradation.

Statistical analysis of the model was performed to evaluate the analysis of variance (ANOVA) using Design Expert® 8.0.0 software (Stat-Ease Inc., Minneapolis, USA).

## Results and discussion

### Synthesis and characterisation of the NH_2_–Fe_x_Cu_y_-MOFs

As a result of the synthesis procedures, four different MOFs have been generated: NH_2_–Fe_0.5_Cu_1_-MOF, NH_2_–Fe_1_Cu_1_-MOF, NH_2_–Fe_1.7_Cu_1_-MOF, and NH_2_–Fe_2.4_Cu_1_-MOF. At first glance (Fig. 1a), all the obtained materials have been powders and the colour differences among them are evident. Clearly, the colour of each material is affected by the proportions of the selected metals, ranging from the brown tone which is primarily caused by Fe to the green tone which is caused by Cu.

The catalyst that best distinguishes itself from the other three synthesised catalysts is NH_2_–Fe_0.5_Cu_1_-MOF, which is the one with the greenest colour due to the high content of Cu with respect to Fe. Of the other three catalysts, NH_2_–Fe_1_Cu_1_-MOF, certain green tones can be detected. Moreover, these tones stand out more when compared to NH_2_–Fe_1.7_Cu_1_-MOF, which already has a completely brown tone, and NH_2_–Fe_2.4_Cu_1_-MOF, which is a much more intense and darker brown colour. This fact has been demonstrated by ICP-OES analysis. Fe and Cu contents are approximately the same as when added in the hydrothermal reactor, resulting in little loss and practically all metals react to form the bimetallic MOFs (Table 2). In addition, the four NH_2_–Fe_x_Cu_y_-MOFs have been analysed to evaluate their physical, chemical, and morphological properties, as described in the next sections.

**Table 2.**
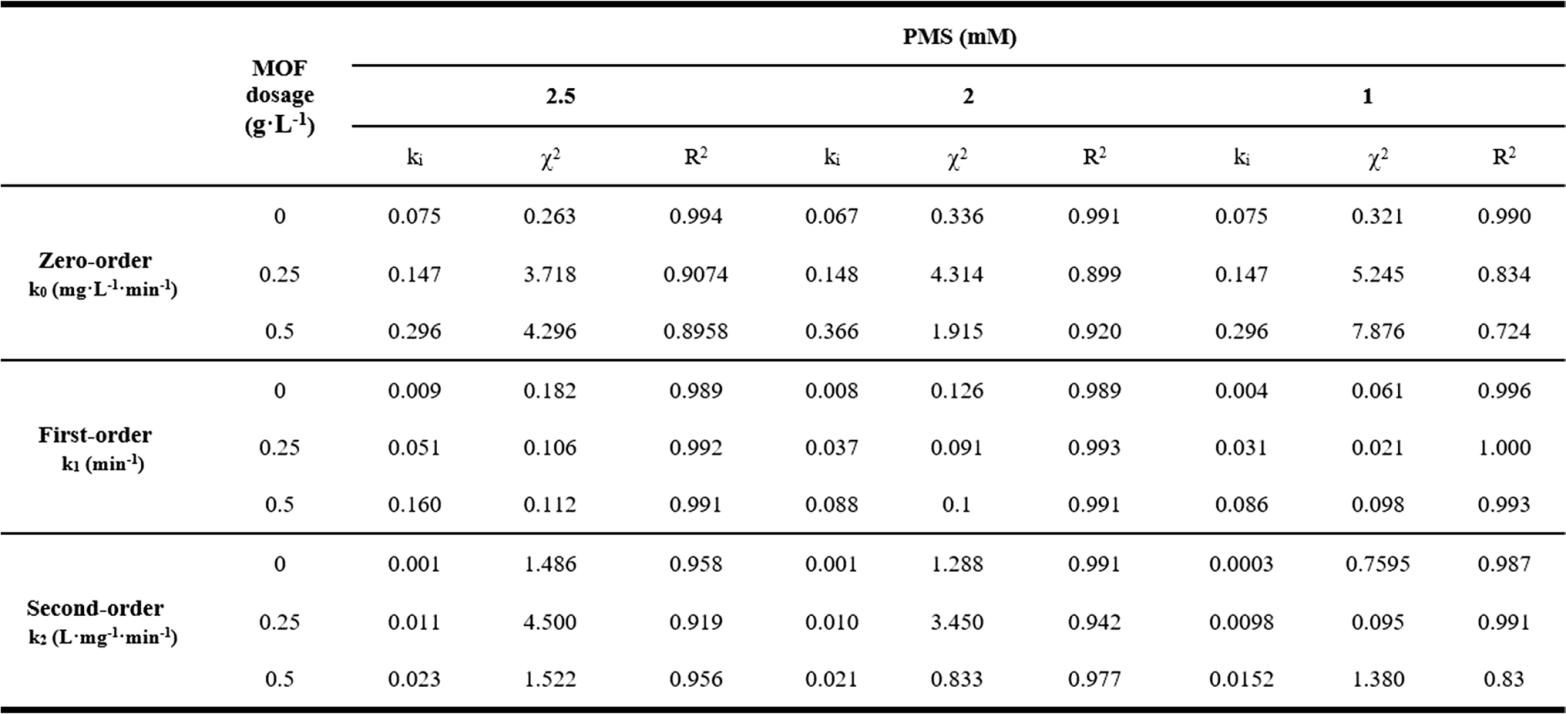
Parameters of the kinetic study for the experiments performed at different NH_2_–Fe_2.4_Cu_1_-MOF dosage and PMS concentration

#### XRD analysis

**Fig. 2:**
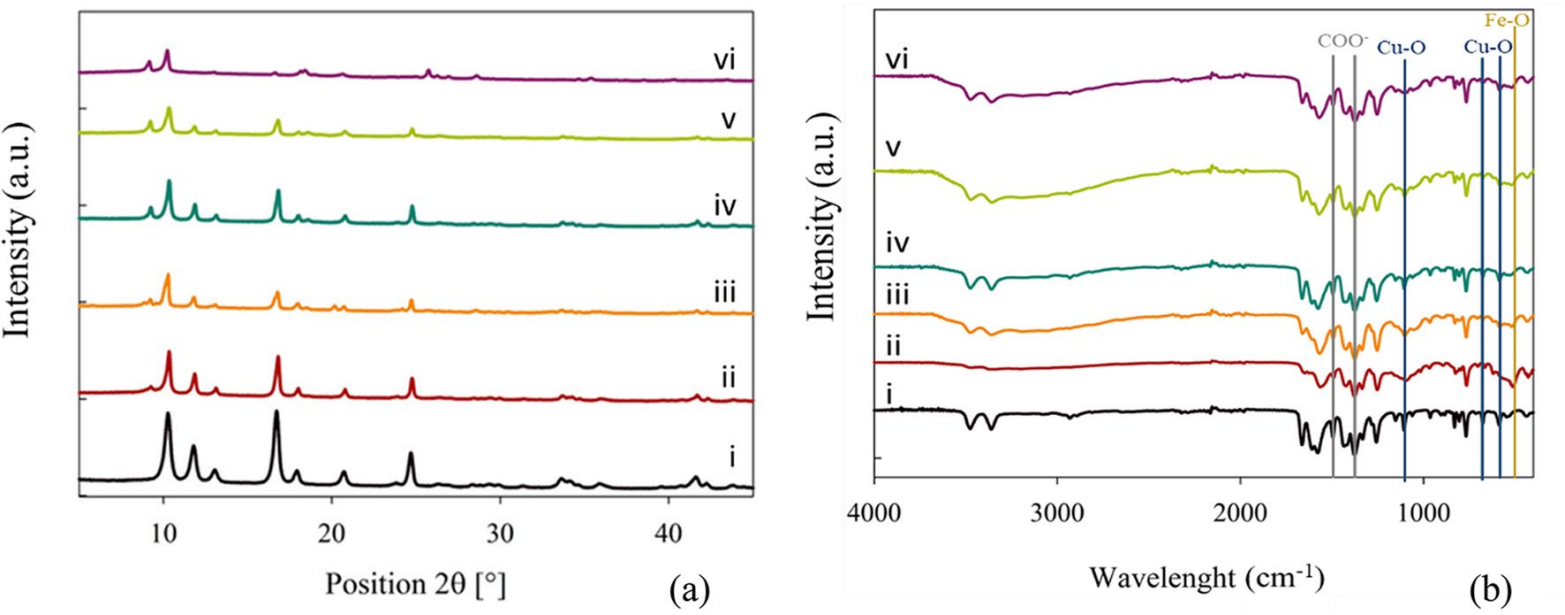
XRD spectra (**a**) and FTIR spectra (**b**) of the bimetallic MOFs and the corresponding NH_2_–Fe-MOF and NH_2_–Cu-MOF. The spectra have been arranged in ascending order of Fe content, starting with NH_2_–Cu-MOF (i, black), NH_2_–Fe_0.5_Cu_1_-MOF (ii, red), NH_2_–Fe_1_Cu_1_-MOF (iii, orange), NH_2_–Fe_1.7_Cu_1_-MOF (iv, blue), NH_2_–Fe_2.4_Cu_1_-MOF (v, green), and NH_2_–Fe-MOF (vi, purple)

From the XRD characterisation, the syntheses of NH_2_–Fe_x_Cu_y_-MOFs have been assured due to their high crystalline structure, as can be seen in Fig. 2a. Depending on the nature of the bimetallic MOF, peaks of different intensities are observed due to the presence of both metals in the structure and the influence of each metal. The spectra of MOFs are clearly differentiated from the spectra of the monometallic MOFs, as NH_2_–Fe-MOF, which is NH_2_-MIL-88B(Fe), and NH_2_–Cu-MOF.

As can be seen from the NH_2_–Fe-MOF spectrum in Fig. 2a, the characteristic peaks are the ones appearing at 2*θ* = 9.2° and 18.5°. The presence of these two peaks has also been reported by Shi et al. (2015) when they synthesised MIL-88B(Fe)MOFs. From their study, it has been found that the XRD pattern of MIL-88B (Fe) and NH_2_-MIL-88B (Fe) show the same XRD pattern, which means that the crystal phase structure is maintained after amine functionalisation. Since there is no change, suggesting that the NH_2_-MIL-88B (Fe) structure is formed by Fe_3_-μ_3_-oxo clusters interconnected by oxidation-stable terephthalate bonds, this interplay should also be reflected in the FTIR spectrum (Fig. 2b). While the reflections presented by the NH_2_-MOF-Cu are the intense reflection in 2*θ* = 11.8° and the weak reflections of 2*θ* = 18.1° and 24.7°. As can be seen, as the proportion of Cu in the bimetallic MOFs decreases, the intensity of these peaks is considerably reduced. In fact, in the comparison of the peak intensities of NH_2_–Fe_0.5_Cu_1_-MOF with NH_2_–Fe_2.4_Cu_1_-MOF, it is observed in the intense reflections of 2*θ* = 11.8° and 16.6° a drop of 38.2% and 30.5%, respectively. This effect also occurs with the increase of Fe in the MOFs, but it is much less pronounced and is mainly seen in the peak at 2*θ* = 9.2°. In fact, if the same comparison is made between NH_2_–Fe_0.5_Cu_1_-MOF and NH_2_–Fe_2.4_Cu_1_-MOF, the increase in the intensity of this peak is only 20% when the Fe content is increased by almost five times. These changes in peak intensity have also been observed in other studies, such as Wu et al. (2023), which synthesised Mn-MOF-74, to which a certain proportion of Fe has been added, in which these changes in peak intensity were observed for the Mn and Fe peaks.

To conclude this characterisation by XRD, it should be noted that in all the XRD spectra of the two types of MOFs, the common peaks are 2*θ* = 10.3°, 13.2°, and 16.6°. These peaks appear with very low intensity in the spectra with high Fe content, such as NH_2_–Fe-MOF, NH_2_–Fe_2.4_Cu_1_-MOF, and NH_2_–Fe_1.7_Cu_1_-MOF, but by superimposing all the spectra, this identification has been perfectly verified. So, it is possible to conclude that all the NH_2_–Fe_x_Cu_y_-MOFs were successfully synthesised and have a particular crystal structure.

#### FTIR analysis

In order to investigate the chemical bonding in the structure, FTIR analysis has been conducted. Figure 2b illustrates that the FTIR spectra are very similar, with only minor modifications, which are also noted. The first remarkable feature is the detection of the abscission peaks of the stretching vibration of the Fe–O bond, which is found at 520 cm^−1^ (Mohebali et al. 2023), and of Cu–O, which is found at 1066 cm^−1^, 676 cm^−1^ and 570 cm^−1^ (Wan et al. 2023), in all the MOFs. Thus, in the MOF structure, the coordination between the two metals is confirmed. Furthermore, in all of them, an intense absorption peak has been found in the 1570 cm^−1^ band, which corresponds to the COO^−^ functional group, which is a group of the NH_2_-BDC ligand. In addition to this intense peak of the COO^−^ group, peaks at 1425 cm^−1^ and 1377 cm^−1^ were detected (Fu et al. 2021). It is evident from the presence of these peaks that the COO^−^ seems that the symmetric tension mode of this group has unfolded, which indicates that this group acts as a bridge, allowing the metals to bind. Similar results had been found by Fdez-Sanromán et al. (2023), who synthesised different types of FeCu-MOF and detected this bridging behaviour of the ligand. Another of the peaks observed in the band at 3500 cm^−1^ and 3200 cm^−1^, corresponds to the N–H and may indicate that the MOF has –NH_2_ functional groups, which is another group from NH_2_-BDC ligand (Yi et al. 2020). Therefore, by means of FTIR analysis, the correct synthesis of MOFs, with the functionalisation with the − NH_2_ group, and the bonds produced in this structure to form the crystalline network have been confirmed once again.

#### Morphological characterisation

**Fig. 3:**
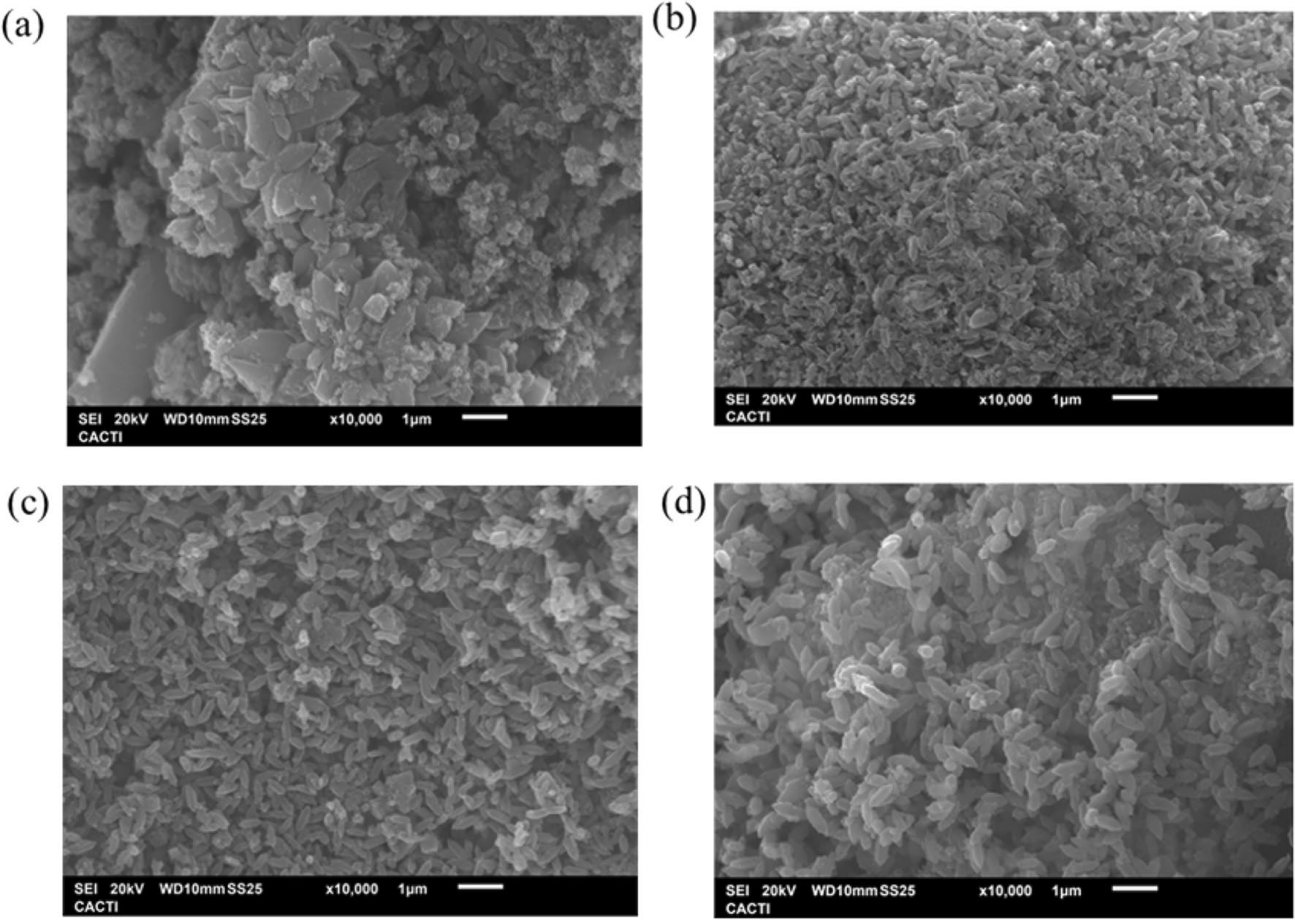
SEM images of the NH_2_–Fe_x_Cu_y_-MOFs: **a** NH_2_–Fe_0.5_Cu_1_-MOF, **b** NH_2_–Fe_1_Cu_1_-MOF, **c** NH_2_–Fe_1.7_Cu_1_-MOF, and **d** NH_2_–Fe_2.4_Cu_1_-MOF

A morphology analysis has been conducted in order to complete the characterisation of the different NH_2_–Fe_x_Cu_y_-MOF (Fig. 3). SEM images reveal that the morphology of the NH_2_–Fe_x_Cu_y_-MOFs is very similar, being predominant the rod shape morphology, and presents a homogeneous distribution and is not agglomerated. As for the MOF shape, this is obtained due to the incorporation of water in the synthesis of this NH_2_–FeCu-MOF, since in the study of Liao et al. (2019), the same Fe-MOF has been synthesised with different ratios of water and DMF and a change in its 3D structure has been denoted, being the mixture with water causing the rod-like shape. In addition, this change in the structure due to the solvent also occurs through other synthesis methods, such as microwave synthesis, as reported Ma et al. (2013). According to their findings, when using the DMF solvent for the synthesis of MIL-88B-Fe, a much wider rod (over 270 nm) was obtained than in the aqueous medium; however, the rod’s length is essentially unchanged. It can be also detected by the images (Fig. 3) that as the proportion of Fe in the NH_2_–FeCu-MOF increases, the rod shape is more evident and has a larger size, with approximately 32.64% increase from NH_2_–Fe_1_Cu_1_-MOF and NH_2_–Fe_2.4_Cu_1_-MOF, whereas when comparing the NH_2_–Fe_1.7_Cu_1_-MOF and NH_2_–Fe_2.4_Cu_1_-MOF, this difference is reduced to only 17.4%. As for the NH_2_–Fe_0.5_Cu_1_-MOF, the size is very similar to that of NH_2_–Fe_2.4_Cu_1_-MOF, having only a difference of 70 µm, but its rod shape is not defined. This fact could be explained by the high Cu content and its tendency to have a rectangular shape as the NH_2_–Cu-MOF.

Thus, based on the characterisation of the four synthesised NH_2_–Fe_x_Cu_y_-MOFs, it can be concluded that these MOFs present good properties to be applied as potential catalysts in AOPs.

### Study of the catalytic activity of different NH_2_–Fe_x_Cu_y_-MOFs

#### Effect of NH_2_–Fe_x_Cu_y_-MOFs composition on the degradation

The catalytic capability of the four synthesised MOFs (NH_2_–Fe_0.5_Cu_1_-MOF, NH_2_–Fe_1_Cu_1_-MOF, NH_2_–Fe_1.7_Cu_1_-MOF, NH_2_–Fe_2.4_Cu_1_-MOF) has been investigated for PMS activation under natural pH. This condition has been selected based on previous studies in which was reported that SO_4_^•−^ is the predominant radical under acid and natural pH ranges by the CuFe-MOF/PMS system (Li et al. 2022; Fdez-Sanromán et al. 2023). In all cases, the PMS concentration has been 1 mM, and the catalyst dosage, 0.2 g·L^−1^.

**Fig. 4:**
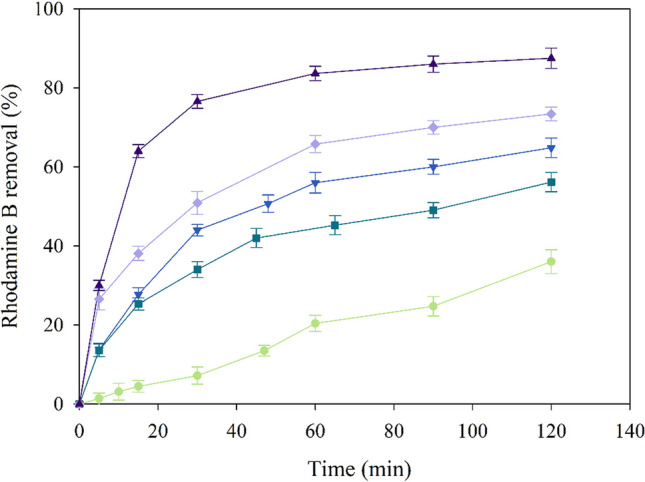
Degradation profiles of the control, with PMS alone (light green, ●) and with the different NH_2_–Fe_x_Cu_y_-MOF: NH_2_–Fe_1_Cu_1_-MOF (cyan, ■), NH_2_–Fe_0.5_Cu_1_-MOF (blue, ▼), NH_2_–Fe_1.7_Cu_1_-MOF (light purple, ♦), NH_2_–Fe_2.4_Cu_1_-MOF (dark purple, ▲)

As can be seen in Fig. 4, the combination of PMS with the different NH_2_–Fe_x_Cu_y_-MOF increases up to 35% of the degradation compared to PMS alone. Only 20% of dye has been removed by PMS within 60 min, indicating that PMS is difficult to self-activation in the atmospheric temperature (Mo and Zhang 2024). This improvement in dye degradation can be attributed to a strong relationship between Fe content in the MOF and a substantial increase has been detected due to Fe concentration. This behaviour has been reported by Tang and Wang (2020), who synthesised different bimetallic MOFs, called Fe_x_Cu_1−x_(BDC) for the degradation by a Fenton process of the pollutant sulfamethoxazole. Mo and Zhang (2024) evaluated the synergistic effect of Fe and Cu determining the followed order: Fe + Cu/PMS > Fe/PMS > Cu/PMS > PMS systems in the rhodamine B. Similar to our study, it can be concluded that, under the working conditions, Fenton-like reactions are favoured and the possible reactions that occur in the media are the following (Eqs. 3–8):

3 $$\equiv \mathrm{Fe}\left(\mathrm{II}\right) + {\mathrm{HSO}}_{5}^{-}\to \equiv \mathrm{Fe}\left(\mathrm{III}\right)+ {\mathrm{OH}}^{-}+ {\mathrm{SO}}_{4}^{\bullet -}$$

4 $$\equiv \mathrm{Fe}\left(\mathrm{III}\right) + {\mathrm{HSO}}_{5}^{-}\to \equiv \mathrm{Fe}\left(\mathrm{II}\right)+ {\mathrm{H}}^{+}+ {\mathrm{SO}}_{5}^{\bullet -}$$

5 $$\equiv \mathrm{Cu}\left(\mathrm{II}\right) + {\mathrm{HSO}}_{5}^{-}\to \equiv \mathrm{Cu}\left(\mathrm{I}\right)+ {\mathrm{OH}}^{-}+ {\mathrm{SO}}_{4}^{\bullet -}$$

6 $$\equiv \mathrm{Cu}\left(\mathrm{I}\right) + {\mathrm{HSO}}_{5}^{-}\to \equiv \mathrm{Cu}\left(\mathrm{II}\right)+ {\mathrm{H}}^{+}+ {\mathrm{SO}}_{5}^{\bullet -}$$

7 $$\equiv \mathrm{Fe}\left(\mathrm{III}\right) + \equiv \mathrm{Cu}\left(\mathrm{I}\right)\to \equiv \mathrm{Fe}\left(\mathrm{II}\right) + \equiv \mathrm{Cu}(\mathrm{II})$$

8 $${\mathrm{SO}}_{4}^{\bullet -}+{\mathrm{H}}_{2}\mathrm{O}\to {\mathrm{H}}^{+}+{\mathrm{SO}}_{4}^{-2} + {\mathrm{OH}}^{\bullet }$$

Monometallic iron- and copper-based materials often display poor ability in the activation of PMS because of the rate-limiting step of Fe (III)/Cu(II) conversion to Fe(II)/Cu(I) (Kim et al. 2018). Thus, several alternatives have been approached for fulfil these drawback such as a Cu-doped CoFe_2_O_4_ nanocatalyst that has been used for PMS activation to remove rhodamine B where the Cu not only activated PMS to produce ROS but also regenerated Co(I) and Fe(II) to accelerate the PMS activation (Mo and Zhang 2024). In our study, it should be noted that NH_2_–Fe_0.5_Cu_1_-MOF has a higher degradation rate due to the higher Cu content and allows the Fenton-like reaction to take place (Eqs. 5 and 6), while NH_2_–Fe_1_Cu_1_-MOF, because it does not have a high Fe and Cu content, these reactions described above, especially reaction (7), do not occur as fast as the other NH_2_–Fe_x_Cu_y_-MOF.

In addition, it is reported in the literature that XRD analysis of the catalyst allows to determine its stability. Thus, a broad peak at 17–39° has been detected by the wide-angle XRD in the patterns of the functionalised silica nanotubes before and after five times of recycling (Aljohani et al. 2023). The XRD patterns of fresh and used CuO–Fe_3_O_4_@C (prepared by thermal conversion of Cu(OAc)_2_/Fe-MOF) indicated that no apparent difference was observed in the crystalline phases of those catalysts and suggested the stability of this catalyst for PMS activation. Accordingly, in our study, the XRD spectra after the treatment showed similar peaks at 10.3–16.6° confirming that NH_2_–Fe_x_Cu_y_-MOF retained their structure after degradation process (Zhu et al. 2023b).

As can be seen in Fig. 4, for the same conditions, the degradation of the dye is increased because of the Fe content in the NH_2_–Fe_x_Cu_y_-MOF. Therefore, NH_2_–Fe_2.4_Cu_1_-MOF has been selected as the best catalyst to use in this study, since it has improved the elimination reaction up to 53% and the rate obtained is 3.7 times higher compared to the use of PMS alone. Since Cu and Fe ions may contribute to PMS activation and are also toxic to a certain degree, the leaching of Cu and Fe ions would be determined after the oxidation reaction. As a result, it has been noted that the concentrations of leached Cu and Fe ions in the solution after 120 min are approximately 0.1–0.18 mg·L^−1^ and 0.05–0.093 mg·L^−1^, respectively. As shown in previous studies (Jiang et al. 2022; Zhu et al. 2023a), the contributions of PMS activation caused by the leached of both metals have been very slight, indicating that there is no obvious effect on the removal of pollutants by the leached ions and PMS system.

#### Study of the influence of the PMS concentration on NH_2_–Fe_2.4_Cu_1_-MOF

**Fig. 5:**
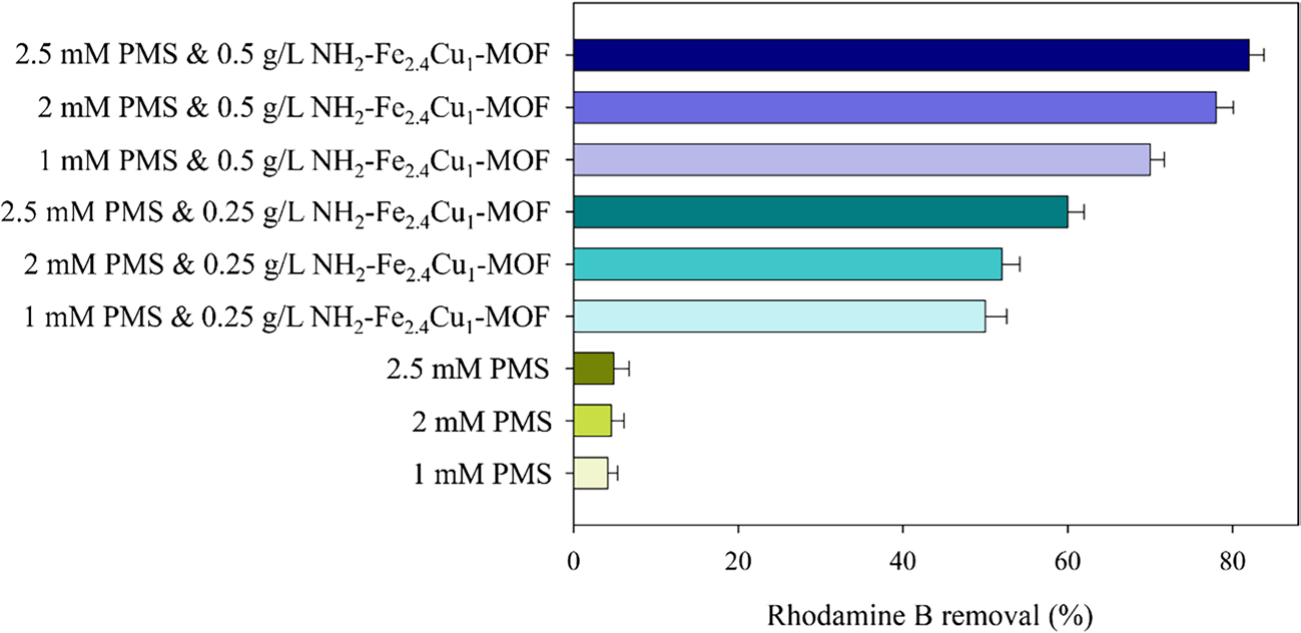
Degradation results of rhodamine B for different concentrations of PMS (1, 2, and 2.5 mM) and NH_2_–Fe_2.4_Cu_1_-MOF dosage (0, 0.25, and 0.5 g·L^−1^) after 15 min. The green bars represent the control experiments, while the light blue bars represent the NH_2_–Fe_2.4_Cu_1_-MOF concentration of 0.25 g·L^−1^ and the purple bars, 0.5 g·L.^−1^

Once the catalyst was selected, the influence of the main factors of the process in the degradation has been tested. Thus, the effect of initial pH, NH_2_–Fe_2.4_Cu_1_-MOF dosage, and PMS concentration for the decolourisation of rhodamine B has been selected and the results were evaluated (Fig. 5).

Since the catalysts have not showed any differences in their catalytic activity, the influence of the initial pH has not been investigated in all assays. This behaviour has also been observed in other studies, such as that conducted by Li et al. (2022), who synthesised a type of FeCu-MOF for PMS activation in order to degrade the dye methylene blue. In all cases, it has been found that when working in a pH range of 3 to 6, no differences in catalytic activity were observed. These results can be justified by the fact that despite starting at different pH, the pH at the end of the reaction is in the range of 2.6–3.5. For this reason, the initial pH was neither included in this study nor in the design of experiments described in the next section.

As is displayed in Fig. 5, the influence of the catalytic activity of NH_2_–Fe_2.4_Cu_1_-MOF is more evident at high catalyst dosages attaining removal values around 70–80%. It should be noted that when performing the control experiments, without using NH_2_–Fe_2.4_Cu_1_-MOF catalyst, no increase in dye removal has been detected, with an elimination rate below 10% in 15 min. Operating at PMS concentration of 1 mM, the reached degradation values have been 5- and 12-fold the reported for the control operating at a catalyst dosage of 0.25 and 0.5 g·L^−1^, respectively. When the PMS concentration was increased from 1 to 2.5 mM, slight improvements were observed at the highest catalyst dosage (0.5 g·L^−1^). Thus, NH_2_–Fe_2.4_Cu_1_-MOF exhibits good catalytic properties to be used as catalyst in a wide range of PMS concentrations and dosages.

According to Lei et al. (2023), when the PMS concentration is increased from 2 to 4 mM, the removal of total petroleum hydrocarbons increases significantly, while if it continues to increase to 8 mM, the degradation effect drops. When the concentration of PMS is too high, a large amount of SO_4_^•−^ would inevitably undergo self-quenching reaction. Thus, it is important to know the effects of PMS and catalyst content on the degradation process, identifying the values at which the self-quenching reaction does not occur.

To characterise the degradation process, kinetic studies for zero-, first-, and second-order reactions were performed. The selection of the best model for fitting degradation experimental data was based on the model corresponding coefficient of determination (*R*^2^) and confirmed by error analysis (chi-square test, *χ*^2^); the lesser the *χ*^2^ value, the better the fit. *χ*^2^ was calculated as follows (Eq. 9):

9 $${\chi }^{2}=\sum_{\mathrm{i}=1}^{\mathrm{n}}\frac{{{(\mathrm{D}}_{\mathrm{e},\mathrm{exp}}-{\mathrm{D}}_{\mathrm{e},\mathrm{cal}})}^{2}}{{\mathrm{D}}_{\mathrm{e},\mathrm{cal}}}$$

where *D*_e,cal_ and *D*_e,exp_ refer to the theoretical and experimental degradation levels, respectively, and *n* is the number of experimental observations. Thus, the degradation profiles during 60 min have been fitted to different models, and the values of kinetic constant, *R*^2^, and *χ*^2^ were calculated (Table 2). The results indicated that the dye removal could be quantitatively described by a first-order kinetic equation (Eq. 10) with respect to the rhodamine B concentration.

10 $${C}_{A}={C}_{A0}\cdot {e}^{-k\cdot t}$$

In Table 2, it is observed a considerable increase in the pseudo-first-order kinetic constant when the PMS concentration was raised, independently of the amount of catalyst considered. It showed a significant impact on degradation as it increased approximately 1.7 times, comparing the results of 0.25 g·L^−1^ catalyst with 1 mM and 2.5 mM. The increase in catalyst dosage at the same PMS concentration also increases the kinetic constant, but at a higher ratio. These results allow the modelling of previous degradation experiments, conducted at different concentrations of PMS and NH_2_–Fe_2.4_Cu_1_-MOF dosages. Therefore, these results confirm the good capability of this MOF for the activation of PMS, showing high catalytic activity in the degradation process of rhodamine B.

**Fig. 6:**
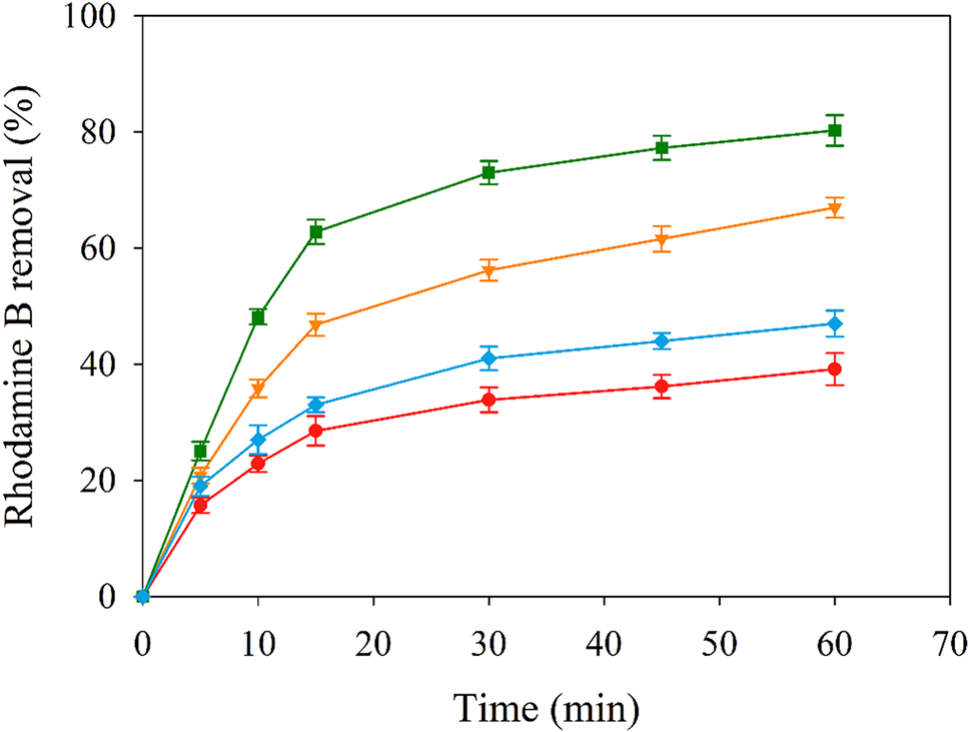
Effect of different scavengers on the removal of rhodamine B to identify radicals: O_2_^•−^ (orange, ▼), SO_4_^•−^ and OH^•^ (blue, ♦), and only OH^•^ (red, ●) and compared with the control experiment (green, ■). The reaction conditions for all experiments were [PMS] = 2.5 mM, [NH_2_–Fe_2.4_Cu_1_-MOF] = 0.25 g·L^−1^, [EtOH] = 500 mM, [p-BQ] = 20 mg·L^−1^, and [TBA] = 500 mM

Activation of PMS to degrade organic compounds is an effective process; however, in this process, it is important to determine whether a radical pathway is used or not. So, quenching assays have been carried out to determine the reaction mechanism by the main ROS and their contribution to the degradation process (Fig. 6). According to the results, the degradation of rhodamine B by the system NH_2_–Fe_2.4_Cu_1_-MOF/PMS involves a radical pathway.

In the presence of EtOH, an effective quencher for SO_4_^•−^ and OH^•^, at a concentration around 120 times that of PMS, a significant reduction in the degradation efficiency of 34% has been detected (Wang et al. 2022). It is noted that the addition of the specific quencher for OH^•^, TBA, did not significantly affect the pollutant removal, with only a 7% of reduction, and, thus, SO_4_^•−^ is dominant for the oxidation process at the operating acidic pH. This fact is in accordance with the literature results, which show SO_4_^•−^ is the main radical present under these conditions (Wang and Wang 2018). Finally, the presence of other radicals such as superoxide O_2_^•−^ has also been detected by p-BQ. Due to the elimination of rhodamine B, the contribution of these radicals is less than previous radicals. Therefore, rhodamine B degradation by the system can be considered a radical pathway, primarily dominated by SO_4_^•−^, OH^•^, and partially by others such as O_2_^•−^.

After determining the great potential of NH_2_–Fe_2.4_Cu_1_-MOF as catalyst, it is necessary to overcome several problems mentioned before. Thus, the utilisation of the NH_2_–Fe_2.4_Cu_1_-MOF, powder-like material, in successive batch treatments or continuous flow systems requires the development of a low-cost and easy operational recovery system. For this purpose, the preparation of the catalyst supported in spheres of PAN polymer was ascertained.

### Synthesis and characterisation of NH_2_–Fe_2.4_Cu_1_-MOF@PAN spheres

To overcome the problems detected when MOFs are used in flow systems, it is proposed the synthesis of the selected MOF over PAN spheres. Among the advantages of the use of this material, it should be highlighted the possibility to obtain particles with different sizes and geometries. Thinking about the use of this catalyst in a packed-bed reactor, spheres of PAN have been used to grow the NH_2_–Fe_2.4_Cu_1_-MOF on their surface. This new catalyst has been denoted as NH_2_–Fe_2.4_Cu_1_-MOF@PAN. The small size of spheres, around 1.68 mm diameter, has been selected to ensure a larger surface area in the reaction and that they are spread homogeneously throughout the reactor.

The obtained material, NH_2_–Fe_2.4_Cu_1_-MOF@PAN, resulted in brown colour spheres from the original ivory colour and a sphere size of around 1.63 mm. According to the BET analysis, the NH_2_–Fe_2.4_Cu_1_-MOF@PAN sphere has a surface area of around 47 m^2^/g that brings on the contact between the metals and the PMS for radical generation. The brown colour of the NH_2_–Fe_2.4_Cu_1_-MOF@PAN spheres is due to the NH_2_–Fe_2.4_Cu_1_-MOF, as can be seen in Fig. 1a and c. Moreover, the colour was homogeneously distributed over the entire surface of the sphere. So, it seems that the NH_2_–Fe_2.4_Cu_1_-MOF is rightfully grown and supported. In addition, acid digestion of NH_2_–Fe_2.4_Cu_1_-MOF@PAN spheres has been performed, and the obtained liquid has been analysed by ICP-OES. The attained results have demonstrated that the ratio Fe:Cu 2.4:1 has been achieved, thus, getting the desired ratio of the metals.

The prepared NH_2_–Fe_2.4_Cu_1_-MOF@PAN spheres have been then characterised by FTIR and SEM to ensure that the catalyst has been supported correctly over the PAN spheres keeping their catalytic activity.

#### FTIR analysis

**Fig. 7:**
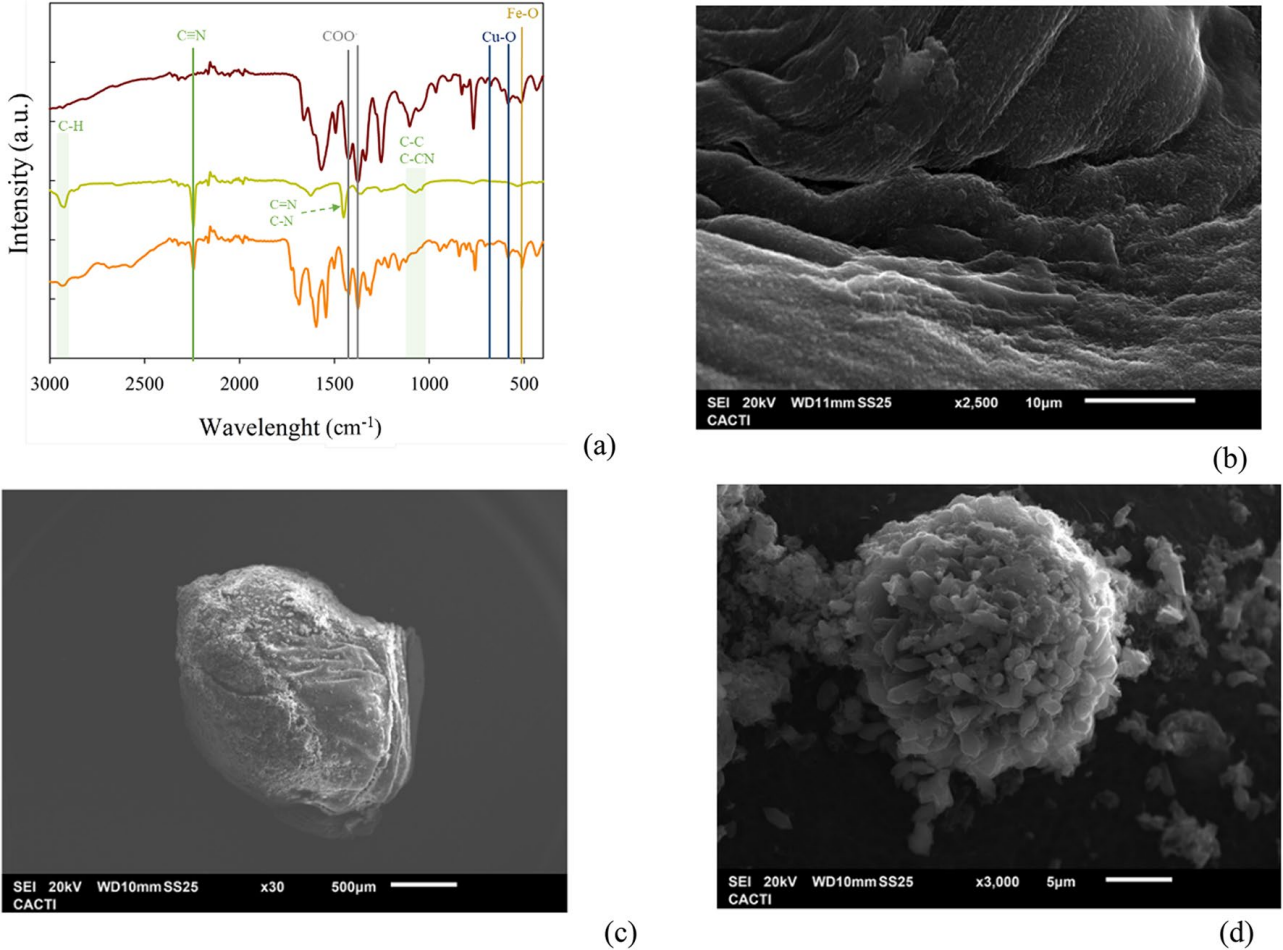
FTIR spectra **a** of NH_2_–Fe_2.4_Cu_1_-MOF (red line), PAN spheres (green line), and NH_2_–Fe_2.4_Cu_1_-MOF@PAN (orange line). SEM images of **b** a section of PAN spheres, and **c** full-size and **d** small sectional from the NH_2_–Fe_2.4_Cu_1_-MOF@PAN sphere

FTIR analysis has been carried out for NH_2_–Fe_2.4_Cu_1_-MOF@PAN spheres, PAN spheres, and the NH_2_–Fe_2.4_Cu_1_-MOF (Fig. 7a). First, the characteristic absorption peaks of the materials alone: PAN spheres and NH_2_–Fe_2.4_Cu_1_-MOF have been identified. From the spectrum of the PAN spheres, the most important absorption peaks are those found at 2243 cm^−1^, which is due to the C≡N-bond, and at 1451 cm^−1^, which is due to the tension occurring between the C–N and C = N-bond (Sun et al. 2020). Other bands of great interest in this spectrum of PAN spheres are the range 2851–2924 cm^−1^, which corresponds to the C–H bond stresses of the CH_2_ and CH_3_ groups, and 1190–1043 cm^−1^, which appears due to the C–C and C–CN bond stresses existing in the PAN monomer (Zhang et al. 2020). Thus, the material preserves its properties also in the spheres. The identified peaks for NH_2_–Fe_2.4_Cu_1_-MOF were already described in the “FTIR analysis” section.

The NH_2_–Fe_2.4_Cu_1_-MOF@PAN spectrum is also displayed in Fig. 7a, that exhibits the characteristic peaks of NH_2_–Fe_2.4_Cu_1_-MOF detected at wavelengths below 1750 cm^−1^, while those related to PAN were identified at longer wavelengths. The most characteristic peaks observed of PAN corresponded to the C–N and C–H tensions; meanwhile, the characteristic MOF peaks found in NH_2_–Fe_2.4_Cu_1_-MOF @PAN were the peaks of the Cu–O, Fe–O, and COO– stresses. It also highlighted the intensity of absorbance peaks, such as those due C≡N. All of them confirmed that there was also a correct coordination in the NH_2_–Fe_2.4_Cu_1_-MOF structure. By identifying the PAN and NH_2_–Fe_2.4_Cu_1_-MOF sphere peaks, it can be concluded that the preparation of this MOF supported on PAN material has been successfully performed. To confirm this, the SEM surface analysis of the NH_2_–Fe_2.4_Cu_1_-MOF @PAN has been performed.

#### Morphological characterisation

The surface study of the PAN spheres and NH_2_–Fe_2.4_Cu_1_-MOF @PAN is displayed in Fig. 7b–d. As can be seen in the figures, a large change in the topography of the PAN sphere before the synthesis of NH_2_–Fe_2.4_Cu_1_-MOF@PAN is observed. The PAN bead has a smooth surface and many folds (Fig. 7b). However, the NH_2_–Fe_2.4_Cu_1_-MOF@PAN sphere has a more spherical shape and fewer folds than before, which may be because the NH_2_–Fe_2.4_Cu_1_-MOF grew in those areas through the linker of the NH_2_–Fe_2.4_Cu_1_-MOF that binds it to the surface of the PAN. This linker function has also been reported by Wu et al. (2017), who synthesised a ZIF-8 supported on a membrane by electrodeposition of the ZnO/2-methylimidazole nanocomposite. Another fact that allows to confirm the formation of NH_2_–Fe_2.4_Cu_1_-MOF@PAN is the roughness observed on the surface (Fig. 7c). With a higher magnification (Fig. 7d) it can be seen the growth in a flower shape of the NH_2_–Fe_2.4_Cu_1_-MOF rods by addition. A similar behaviour was also reported in the literature by Cao et al. (2021), who synthesised a MOF-based Pd@UiO-66-NH_2_@ZnIn_2_S_4_, resulting in a three-dimensional flower-like structure. Also, the EDS analysis confirmed the presence of Fe and Cu in the spheres at the desired proportion. In conclusion, the successful synthesis of NH_2_–Fe_2.4_Cu_1_-MOF@PAN by the solvothermal process has been confirmed.

### Study of the degradation process via PMS activation by NH_2_–Fe_2.4_Cu_1_-MOF@PAN spheres

#### Preliminary studies

**Fig. 8:**
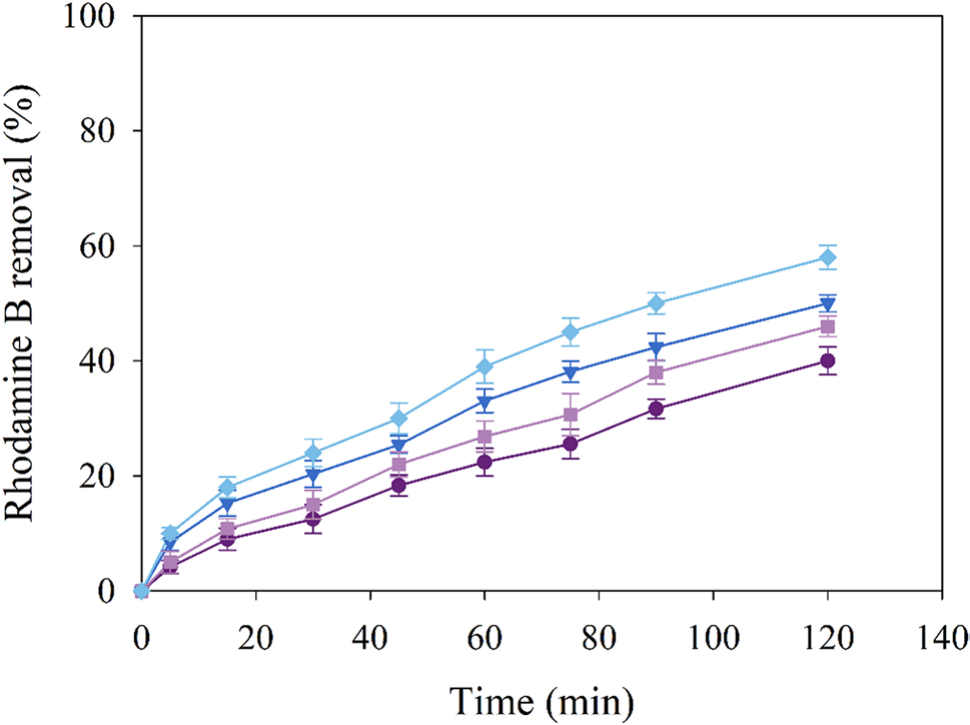
Degradation profiles of rhodamine B by PMS activation by NH_2_–Fe_2.4_Cu_1_-MOF@PAN, at natural pH, at different PMS concentrations and NH_2_–Fe_2.4_Cu_1_-MOF@PAN dosages. The blue tones of the lines represent the use of 0.5 g/L NH_2_–Fe_2.4_Cu_1_-MOF@PAN for a PMS concentration of 2 mM (♦) and 1 mM (▼), meanwhile, the purple tones, 0.3 g/L of NH_2_–Fe_2.4_Cu_1_-MOF@PAN and the PMS concentrations are 1 mM (●) and 2 mM (■)

After confirming that the supported catalyst preparation has been performed correctly, the catalytic capability of NH_2_–Fe_2.4_Cu_1_-MOF@PAN for PMS activation has been evaluated in the rhodamine B degradation. An initial screening at different concentrations of PMS and NH_2_–Fe_2.4_Cu_1_-MOF@PAN dosage has been carried out and the obtained results are shown in Fig. 8.

The selected concentration of PMS and NH_2_–Fe_2.4_Cu_1_-MOF@PAN dosage, like the one obtained in the “Study of the catalytic activity of different NH_2_–Fe_x_Cu_y_-MOFs” section for NH_2_–Fe_2.4_Cu_1_-MOF, did not allow to keep the same ratios and the differences in the removal percentages have been smaller. It can be observed that there are relationships among the different variables in the range of conditions evaluated. Operating at the same PMS concentration, the increase of the NH_2_–Fe_2.4_Cu_1_-MOF@PAN can allow approximately 15% improvement to the process. Similarly, increasing the concentration of PMS shows a similar result where the improvement is approximately the same, with about a 16% increase in rhodamine B removal. Thus, to quantify the relationship among the different variables, a design of the experiments has been performed for the degradation level of rhodamine B.

Summarising up, it is necessary to use high concentrations of PMS and NH_2_–Fe_2.4_Cu_1_-MOF@PAN to achieve degradation levels like the previously attained for NH_2_–Fe_2.4_Cu_1_-MOF.

#### Design of experiments of the degradation process via NH_2_–Fe_2.4_Cu_1_-MOF@PAN spheres

In order to establish the relationships among the operational variables and their optimisation, a design of experiments (2^3^), with time (*X*_1_), NH_2_–Fe_2.4_Cu_1_-MOF@PAN dosage (*X*_2_), and PMS concentration (*X*_3_) as variables and the elimination of rhodamine B as the response function (Table 1) has been carried out. NH_2_–Fe_2.4_Cu_1_-MOF@PAN dosage is considered in a range of 0.25 to 1.25 g·L^−1^, and PMS of 1.5 to 2.5 mM. In addition, it should be noted that the reaction time will also be evaluated as a variable of the experimental design because, when working with the free catalyst, fast degradation of the dye has been detected. Therefore, the range that has been established is from 10 to 90 min. The experimental results have been fitted to a quadratic model and the ANOVA analysis of this fit is shown in Table 3.

**Table 3:** ANOVA analysis for CCD-FC response surface from the quadratic model fit

*Source*	*Sum squares*	*Degree freedom*	*Mean square*	*F-value*	*Prob* > *F*
Model	7925.4758	9	880.6084	82.0938	< 0.0001
X_1_-PMS	942.7068	1	942.7068	87.8828	< 0.0001
X_2_-NH_2_–Fe_2.4_Cu_1_-MOF@PAN	283.1885	1	283.1885	26.3999	0.0004
X_3_-time	6132.1236	1	6132.1236	571.6607	< 0.0001
X_1_ X_2_	8.6978	1	8.6978	0.8108	0.3890
X_1_ X_3_	337.4632	1	337.4632	31.4596	0.0002
X_2_ X_3_	70.3493	1	70.3493	6.5582	0.0283
X_1_^2^	66.3951	1	66.3951	6.1896	0.0321
X_2_^2^	86.3683	1	86.3683	8.0516	0.0176
X_3_^2^	15.6860	1	15.6860	1.4623	0.2544
Std. dev	3.28		*R*-squared		0.9866
Mean	33.85		Adj *R*-squared		0.9746
C.V. %	9.68		Pred *R*-squared		0.9640
			Adeq precision		34.37

The model resulted as significant which verified the validity of the obtained model. Furthermore, it is important to ensure that the lack of fit *F*-value is non-significant to ensure that the model is well fitted. As this parameter had a value of 0.3, it is a non-significant term, so there is an 89.31% chance that some of the *F*-values found in this model are due to noise rather than lack of fit. Summarising the results of the analysis, the factors presenting significant effects are *X*_1_, *X*_2_, and *X*_3_ and the interaction effects *X*_1_*X*_3_, *X*_2_*X*_3_, *X*_1_^2^, and *X*_2_^2^ resulted to be also significant. In fact, when the *p*-value is less than 0.001, it means that they are very significant parameters in the obtained model. While the *p*-values greater than 0.1, they refer to the non-significant terms, which in this case are *X*_1_*X*_3_ and *X*_3_^2^. There is good agreement between the predicted *R*^2^ (0.964) and the adjusted *R*^2^ (0.975), between the theoretical and experimental values. Furthermore, another point to highlight from Table 3 is the value of Adeq Precision, which is a term that is a ratio between the signal and the noise. In this case, this value is 34.37, which is 8.59 times higher than the minimum ratio which must be taken into consideration that the ratio between these terms is adequate. Therefore, the conclusion is that with this mathematical adjustment to a quadratic model, it is possible to navigate over the space designed by the variables. The mathematical equation of the model in terms of coded factors is shown in Eq. (10).

10 $$\mathrm{Rhodamine\;B\;removal\;}\left(\mathrm{\%}\right)\;=\;35.39 + 9.71\cdot {X}_{1} + 9.71\cdot {X}_{2}+24.76\cdot {X}_{3}-1.04 \cdot {X}_{1}\cdot {X}_{2}+6.49 \cdot {X}_{1}\cdot {X}_{3}+2.97 \cdot {X}_{2}\cdot {X}_{3}+4.91\cdot {X}_{1}^{2}-5.60\cdot {X}_{2}^{2}-2.39\cdot {X}_{3}^{2}$$

As can be seen from Eq. (10), the parameters that are significant to the model are those that have a larger coefficient, so with small changes in this value, the response undergoes large changes. Furthermore, with Eq. (10), the effect of the variable on the process can be deduced.

**Fig. 9:**
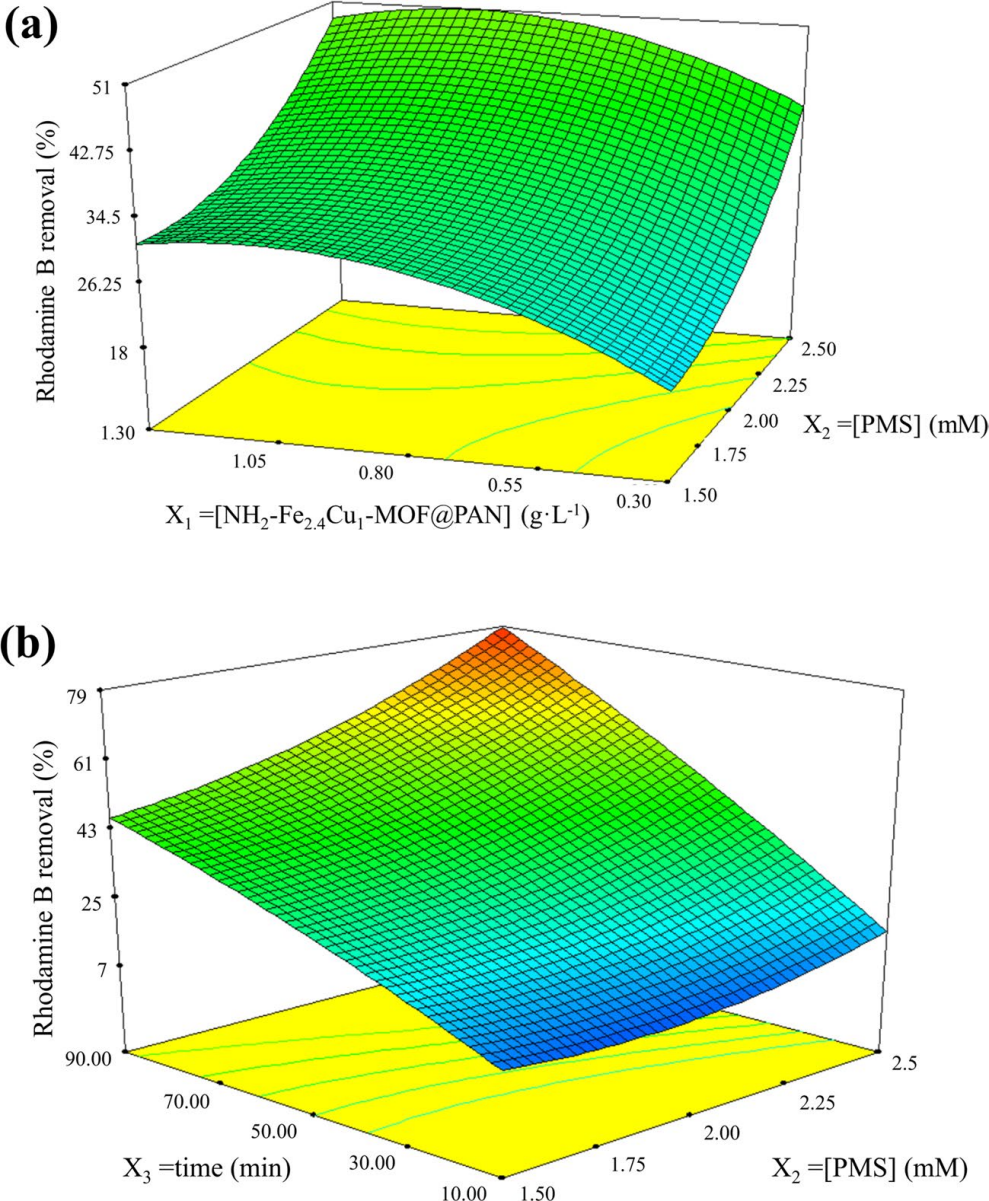
3D representation of response surfaces **a** for the interaction of concentration of PMS and catalyst and **b** for the time and PMS concentration

In addition, the 3-dimensional response surfaces shown in Fig. 9a and b display how the variables of PMS concentration and NH_2_–Fe_2.4_Cu_1_-MOF@PAN dosage (Fig. 9a) and the variable time and PMS concentration (Fig. 9b) affected the whole region that was limited in the 3D cube.

#### Optimisation, validation, and reuse of NH_2_–Fe_2.4_Cu_1_-MOF@PAN spheres

Based on the previous design of experiments, the determination of the optimal conditions to maximise the degradation has been carried out. From the model optimisation, the maximum pollutant degradation is achieved at 2.5 mM of PMS and 90 min with 1.19 g·L^−1^ of NH_2_–Fe_2.4_Cu_1_-MOF@PAN, attaining a theoretical value of 80.92% which has been validated experimentally. From this validation, a degradation of 80.8% has been obtained, so the percentage difference between the theoretical and the real value is only 0.14%. Therefore, it can be remarked that this quadratic model fits correctly with the tested process.

**Fig. 10:**
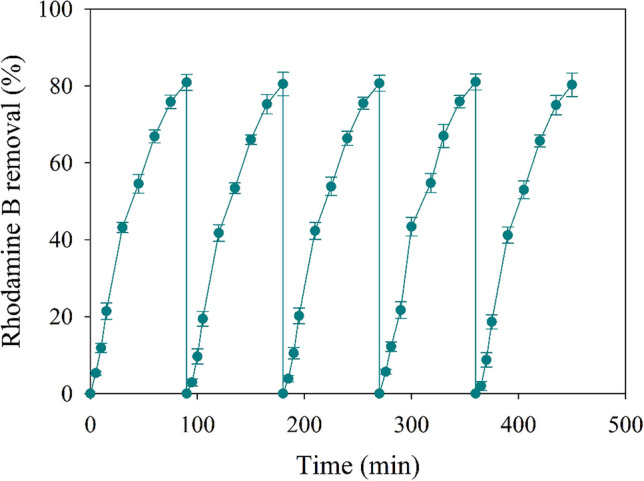
Degradation profiles of rhodamine B over the five cycles using the optimal conditions of the Fenton-like process (2.5 mM PMS, 90 min of time reaction and 1.19 g·L.^−1^ NH_2_–Fe_2.4_Cu_1_-MOF@PAN)

Since the main interest of this supported material is whether it can be applied in continuous processes, a series of tests were carried out with NH_2_–Fe_2.4_Cu_1_-MOF@PAN spheres as catalysts in successive cycles. Therefore, as can be seen in Fig. 10, five cycles have been carried out under the optimum conditions obtained previously. In these cycles, it was evaluated the NH_2_–Fe_2.4_Cu_1_-MOF@PAN spheres’ stability and effectiveness for the activation of PMS.

The degradation profiles in the five cycles are quite similar with less than 5% of reduction in the removal efficiency. Several studies using composite materials achieved similar results. Thus, after 5 cycles, the degradation efficiency of methyl orange by a magnetic CuFe_2_O_4_/zeolite composite was declined 1.6% (Wang et al. 2023a) or 2CuO@1Fe-MFI zeolite that in comparison to impregnation, encapsulation gives rise to the better catalytic degradation activity as well as the better reusability with removal rate constants almost constant value for five cycles (Zou et al. 2023).

In addition, elemental analysis of the Fe and Cu metals in the liquid phase has been carried out for each cycle to determine possible leaching of the materials, and it was found that the release of these metals is minimal, less than 3%, which explains the small variation in the dye removal efficiency. Therefore, in addition to having successfully synthesised NH_2_–Fe_2.4_Cu_1_-MOF on these PAN spheres, it can be stated that it is a feasible catalyst with good stability and effectiveness.

## Conclusions

In this research, a one-pot solvothermal process for the synthesis of bimetallic MOFs with different Fe/Cu ratios has been developed. The feasibility of the synthesised NH_2_–Fe_x_Cu_y_-MOFs as a catalyst in the activation of PMS for the degradation of rhodamine B has been proven. The best degradation levels have been obtained using NH_2_–Fe_2.4_Cu_1_-MOF, and it has been found a significant effect of the bimetallic MOF iron content.

To address the operational problems detected due to the small size of MOFs, the synthesis of catalysts by growing the NH_2_–Fe_2.4_Cu_1_-MOF on PAN spheres has been proposed and determined as a viable alternative. The new catalyst, NH_2_–Fe_2.4_Cu_1_-MOF@PAN, has been successfully synthesised with high physical stability. Moreover, the optimal conditions of the system NH_2_–Fe_2.4_Cu_1_-MOF@PAN/PMS for maximising rhodamine B degradation have been ascertained and validated. Additionally, the quenching test indicated that SO_4_^•−^ and OH^•^ have been the dominant active species that participated in the catalytic oxidation reaction.

Finally, it should be noted that this study may represent a breakthrough in AOPs since a robust heterogeneous catalyst system, NH_2_–Fe_2.4_Cu_1_-MOF@PAN/PMS, with high reusability and without losing its activity in successive cycles, has been developed. This study provides a feasible and promising strategy for the preparation of an excellent and environmentally friendly catalyst that can be applied to scaling up and operating in continuous flow systems.

## Data Availability

The datasets used and analysed during the current study are available from the corresponding author on reasonable request.
